# Health literacy as a mediator between work-family conflict and anxiety and depressive symptoms among white- and blue-collar workers in China

**DOI:** 10.3389/fpubh.2026.1824933

**Published:** 2026-06-08

**Authors:** Junfeng Yuan, Lin Luo

**Affiliations:** 1 School of Physical Education, Guizhou Normal University, Guiyang, Guizhou, China; 2 Key Laboratory of Brain Function and Brain Disease Prevention and Treatment of Guizhou Province, Guiyang, Guizhou, China

**Keywords:** anxiety symptoms, depressive symptoms, family-to-work conflict, health literacy, occupational differences, white- and blue-collar workers, work-to-family conflict

## Abstract

**Background:**

Work-family role conflict is a prevalent source of stress among working adults and has been consistently associated with anxiety and depressive symptoms. Health literacy, which refers to an individual's capacity to obtain, comprehend, and utilize health-related information to make informed decisions, may serve as a crucial mediator in the relationship between work-family conflict and anxiety and depressive symptoms. Furthermore, workers in different occupational categories may differ substantially in their exposure to and coping mechanisms for such conflicts.

**Objective:**

To examine whether the three components of health literacy—health promotion, disease prevention, and health care—mediate the associations of work-to-family conflict (WFC) and family-to-work conflict (FWC) with anxiety and depressive symptoms among white- and blue-collar workers, and to explore differences across occupational groups.

**Methods:**

Data were drawn from the 2021 Psychology and Behavior Investigation of Chinese Residents. A cross-sectional survey was conducted among 4,591 working adults, including 2,487 white-collar and 2,104 blue-collar employees. Data were collected on sociodemographic characteristics, WFC, FWC, health literacy, and anxiety and depressive symptoms assessed using validated instruments. Group comparisons, partial correlation analysis, and bootstrap-based parallel mediation modeling were employed.

**Results:**

Blue-collar workers reported significantly higher FWC scores (*p* = 0.003) and lower health literacy across all components (*p* < 0.001) compared to white-collar workers. No significant occupational differences were observed in overall anxiety or depressive symptom scores. WFC and FWC were positively associated with anxiety and depressive symptoms in both groups; however, the indirect associations through health literacy dimensions differed. Among white-collar workers, disease prevention literacy mediated the associations of WFC and FWC with depressive symptoms. Among blue-collar workers, health care literacy mediated the associations of WFC and FWC with anxiety and depressive symptoms, while health promotion literacy additionally mediated the association between WFC and depressive symptoms. Additionally, the negative correlation between WFC and health literacy was stronger in blue-collar workers, whereas the positive correlation between FWC and anxiety and depressive symptoms was more pronounced in white-collar workers.

**Conclusion:**

Occupational differences may partly characterize the mediating pathways between health literacy and anxiety and depressive symptoms in the context of work-family conflict. Workplace health promotion programs for white-collar workers should emphasize strengthening disease prevention literacy, while strategies for blue-collar workers should aim to concurrently enhance skills in health care and health promotion. Tailoring workplace health programs to occupational characteristics may provide more effective means of addressing anxiety and depressive symptoms associated with work-family role conflicts. Given the cross-sectional design, these findings should be interpreted as associations rather than evidence of causal pathways.

## 1 Introduction

With China's accelerated economic development and the intensification of workplace competition, employees across diverse industries are subjected to mounting occupational stress. Although occupational stress is not a direct cause of specific occupational diseases, its long-term accumulation may exert chronic effects through non-specific physiological mechanisms, ultimately being associated with poorer physical health and psychological wellbeing (1–3). According to the stress-coping theory proposed by Lazarus and Folkman (1984), persistent stress that exceeds an individual's coping capacity may be linked to poorer physical and psychological wellbeing. This theoretical foundation helps explain why a wealth of empirical studies has shown that chronic work stress not only impairs emotional regulation systems but also disrupts physiological functioning, thereby being associated with greater anxiety and depressive symptoms (4–7). In China, mental health problems among working adults have become an increasingly important public health concern. Rapid socioeconomic transformation, intensified workplace competition, long working hours, job insecurity, and growing family caregiving responsibilities may jointly increase psychological strain among employees. Anxiety and depressive symptoms are two common psychological symptom outcomes in working populations and are closely related to impaired work performance, reduced quality of life, increased health service use, and productivity loss. Therefore, examining the factors associated with anxiety and depressive symptoms among Chinese workers is important not only for occupational health but also for broader public health promotion.

Among the various sources of occupational stress, work-family conflict (WFC) has been identified as a particularly salient challenge for contemporary professionals and is consistently associated with anxiety and depressive symptoms (8, 9). WFC refers to the inter-role tension and conflict experienced when individuals attempt to balance work and family role demands (10). Based on the role conflict theory proposed by Kahn et al. (1964), psychological stress may emerge when the demands of work and family roles are incompatible. Accordingly, researchers have classified WFC into two distinct forms: work-to-family conflict (WFC), in which work demands interfere with family responsibilities—such as extended working hours reducing time for family (11); and family-to-work conflict (FWC), in which family obligations hinder work performance—such as family issues diminishing attention or productivity at work (12).

Building upon role conflict theory, WFC primarily stems from excessive job demands, such as overtime or scheduling conflicts, which prevent individuals from adequately fulfilling their family roles (13). Research has demonstrated strong associations between WFC and negative psychological outcomes, including mental fatigue, anxiety, and depressive symptoms (14), as well as reduced life satisfaction and overall wellbeing (15). In contrast, FWC arises from family responsibilities—such as child-rearing and household chores—that interfere with work performance, and has been associated with elevated psychological strain and reduced work efficiency (16), as well as lower family satisfaction (17, 18). Notably, studies indicate that WFC is more strongly associated with work-related outcomes, including job burnout and decreased job satisfaction (19), whereas FWC is more closely associated with family-related outcomes, including the quality of family relationships and family cohesion (20). Hobfoll's Conservation of Resources Theory (1989) provides a robust theoretical framework for understanding this phenomenon—work-family conflict represents a process of resource depletion, whereby the excessive consumption of limited personal resources (e.g., time, energy, attention) may be associated with higher levels of anxiety and depressive symptoms.

A large body of international and Chinese empirical evidence has confirmed a significant association between work-family conflict and anxiety and depressive symptoms, particularly under high-pressure working conditions (9, 21). In China, the widespread adoption of high-intensity work models such as the “996” schedule (9 a.m. to 9 p.m., six days a week) may intensify the imbalance between work and family, thereby being linked to poorer psychological well-being (22) (Zheng & Qiu, 2023). According to Clark's (2000) Work-Family Border Theory, individuals must establish and manage boundaries between the work and family domains, each of which has its own rules and expectations. The capacity to effectively manage these boundaries becomes particularly critical in high-stress environments. Although existing research has made strides in elucidating the direct associations between work-family conflict and anxiety and depressive symptoms, significant gaps remain in exploring mediating mechanisms, especially those involving protective factors. Furthermore, the lack of representative sampling and insufficient examination of occupational differences in the role of health literacy—an emerging protective resource—limits the generalizability and depth of current findings (23–25). These limitations are particularly relevant in the Chinese context, where white- and blue-collar workers may differ substantially in job demands, schedule flexibility, socioeconomic resources, health information access, and coping opportunities. Therefore, examining occupational differences may help clarify whether the associations linking work-family conflict, health literacy, and anxiety and depressive symptoms operate through the same or different mechanisms across worker groups.

Health literacy, defined as the ability to obtain, understand, and apply health information to maintain and promote health (26), has garnered increasing attention in the field of modern health promotion. High levels of health literacy are associated with improved capacity to manage stress and conflict, thereby serving as a key protective factor in the maintenance of psychological wellbeing (27, 28). From the perspective of Bandura's (1986) Social Cognitive Theory, health literacy may improve self-efficacy, shaping how individuals appraise and cope with work-family conflict. This, in turn, may buffer its adverse associations with anxiety and depressive symptoms. By improving health literacy, individuals may become more adept at managing the stress associated with work-family conflict, thus showing lower levels of anxiety and depressive symptoms (29, 30). Health literacy may also operate as a mediating mechanism rather than only as a general protective resource. Work-family conflict may be associated with reduced time, energy, and cognitive resources, thereby limiting individuals' ability to seek health information, engage in preventive behaviors, use health services appropriately, and maintain health-promoting routines. Lower health literacy may then be linked to greater vulnerability to anxiety and depressive symptoms. From this perspective, health literacy provides a theoretically plausible pathway linking work-family conflict to anxiety and depressive symptoms.

**Figure 1:**
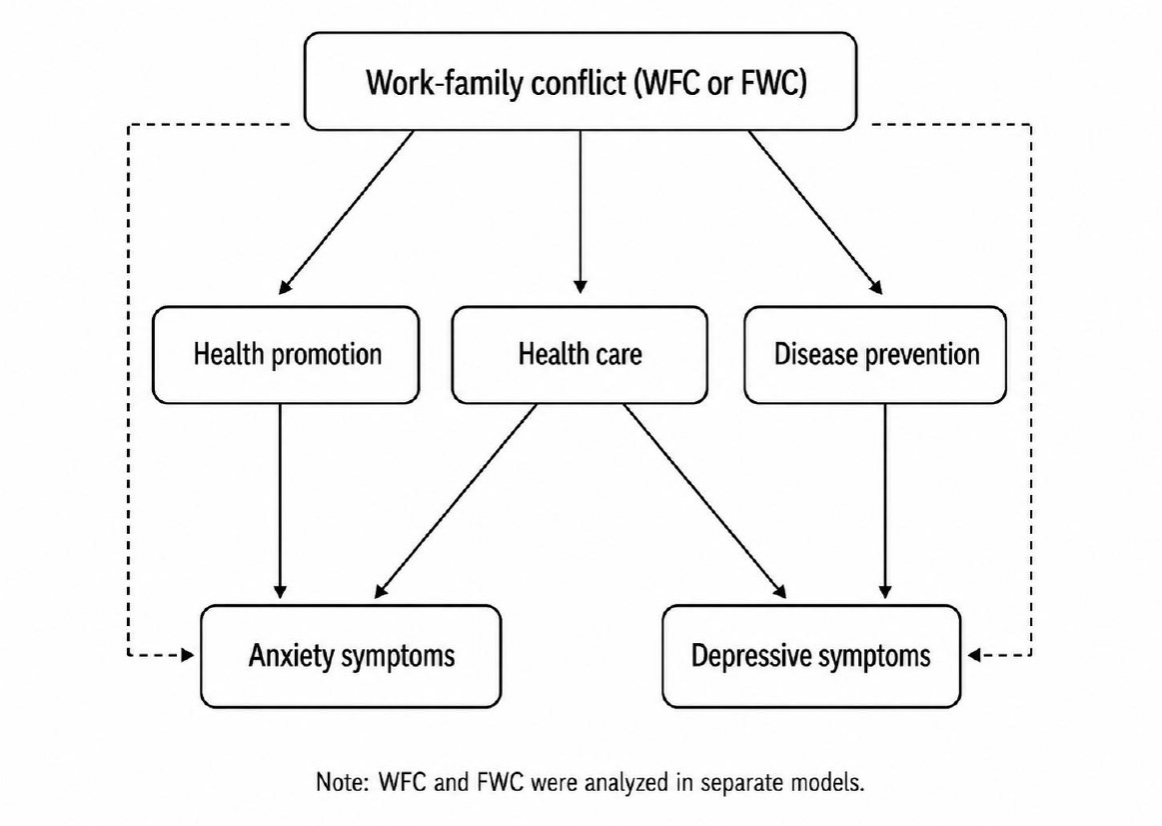
Conceptual framework of the mediation model.

Given this context, the present study employs nationally representative data to examine whether health literacy is associated with the relationships between work-to-family and family-to-work conflict and anxiety and depressive symptoms. Specifically, the study investigates the mediating role of health literacy in the relationship between work-family conflict and anxiety and depressive symptoms, with the goal of informing evidence-based strategies to improve psychological wellbeing among white- and blue-collar workers. This study focuses on three dimensions of health literacy—health promotion, health care, and disease prevention—and compares their mediating roles across occupational groups. By doing so, the study aims to clarify whether different components of health literacy explain the associations of WFC and FWC with anxiety and depressive symptoms differently among white- and blue-collar workers (Figure 1). Given the cross-sectional design, the proposed framework is interpreted as an association-based conceptual model rather than a causal pathway.

## 2 Participants and methods

### 2.1 Study participants

This study utilized data from the 2021 wave of the Psychology and Behavior Investigation of Chinese Residents (PBICR) (31). PBICR is a large-scale, nationwide survey designed to systematically collect high-quality micro-level data from the general population, targeted subgroups, and patients with specific conditions. Its aim is to investigate the psychosocial determinants of health among Chinese residents and to support interdisciplinary research across the life course. The 2021 PBICR covered 34 provinces and 120 cities across China, offering strong national representativeness and the ability to capture psychological and behavioral characteristics across regions, age groups, and genders. The survey adopted a multistage, stratified, community-based sampling strategy. Provinces and cities were first selected to ensure broad geographic coverage, and designated community survey sites were then established with the assistance of local community health centers or neighborhood committees. Eligible residents were recruited according to predefined inclusion and exclusion criteria. This sampling procedure was designed to improve the demographic and geographic representativeness of the survey population. Ethical approval for PBICR was granted by two institutional review boards (Approval numbers: JKWH-2021-01; JNUKY-2021-018), and the study strictly adhered to the ethical principles of the Declaration of Helsinki. Eligible participants were required to possess the capacity to comprehend and complete the survey independently or with assistance, have no apparent cognitive impairments, and provide informed consent. The survey included multidimensional indicators such as sociodemographic characteristics, family background, and psychological symptom status. A total of 11,031 valid responses were collected. For this study, we selected working individuals aged 18–60 based on the Occupational Classification of the People's Republic of China. After excluding logically inconsistent or incomplete data, a final sample of 4,591 valid cases was retained (2,382 females and 2,209 males). Participants were classified as white-collar workers (primarily engaged in neurocognitive activities) or blue-collar workers (primarily engaged in muscular activities) based on the nature of their occupations.

### 2.2 Study measures

#### 2.2.1 Data collection

The survey collected general demographic information, including age (categorized as 19–30, 31–40, 41–50, and 51–60 years), gender (female and male), marital status (married vs. unmarried, with the latter including single, divorced, and widowed individuals), education level (junior high school or below, high school/vocational school, college/university, master's/doctoral degree), current occupation (classified as white- or blue-collar), monthly per capita household income ( ≤ 3,000 RMB, 3,001–7,500 RMB, ≥7,501 RMB), household registration type (agricultural vs. non-agricultural), and place of residence (rural or urban). Health-related indicators included presence of chronic disease, smoking status, and alcohol consumption (each dichotomized as yes/no). These variables were treated as potential confounders in the subsequent partial correlation and mediation analyses because they may be associated with occupational category, work-family conflict, health literacy, and mental health outcomes.

#### 2.2.2 Work-to-family and family-to-work conflict

Work-family conflict was assessed using the Work and Family Conflict Scale (WAFCS) (8, 12). The scale includes 10 items divided into two subscales: work-to-family conflict (WFC) and family-to-work conflict (FWC). The WFC subscale measures the extent to which work interferes with family life (e.g., “My job prevents me from spending enough time with my family”), while the FWC subscale assesses how family responsibilities disrupt work performance (e.g., “My family obligations leave me too exhausted to focus at work”). Items were rated on a five-point Likert scale ranging from 1 (“strongly disagree”) to 5 (“strongly agree”), with higher scores indicating greater perceived conflict. In this study, Cronbach's α coefficients were 0.910 for the WFC subscale and 0.935 for the FWC subscale, indicating excellent internal consistency.

#### 2.2.3 Health literacy

Health literacy was assessed using the Short-Form Health Literacy Scale developed by Sun Xiaonan, Chen Ke, Wu Yunchou, et al. (32). This scale was developed based on classical test theory and item response theory and is designed to provide a concise assessment of individuals' health literacy. The scale covers three dimensions: health promotion, health care, and disease prevention. The health promotion dimension reflects individuals' ability to obtain, understand, and apply information related to healthy lifestyles and health-promoting behaviors. The health care dimension reflects the ability to understand and use information related to health services, medical consultation, and appropriate health-seeking behavior. The disease prevention dimension reflects the ability to recognize disease risks, understand preventive information, and adopt preventive health behaviors. Higher scores indicate higher levels of health literacy.

The scale has demonstrated good construct validity in previous validation research. Confirmatory factor analysis showed acceptable model fit, with χ^2^/df = 10.844, goodness-of-fit index (GFI) = 0.985, adjusted goodness-of-fit index (AGFI) = 0.971, normed fit index (NFI) = 0.986, comparative fit index (CFI) = 0.987, and root mean square error of approximation (RMSEA) = 0.051. In the present study, the total health literacy score and the three dimensional scores were used in the analysis. The Cronbach's α coefficient for the total health literacy scale was 0.913, indicating good internal consistency.

#### 2.2.4 Depressive symptoms

Depressive symptoms were measured using the Patient Health Questionnaire-9 (PHQ-9) (33), a widely used self-report tool for screening and assessing the severity of depression. The PHQ-9 is based on the diagnostic criteria for major depressive disorder in the Diagnostic and Statistical Manual of Mental Disorders, Fourth Edition (DSM-IV), and comprises nine items capturing core symptoms such as depressed mood, anhedonia, sleep disturbances, fatigue, changes in appetite, feelings of worthlessness, concentration difficulties, psychomotor changes, and suicidal ideation. Responses were rated on a four-point Likert scale according to symptom frequency over the past two weeks, ranging from 0 (“not at all”) to 3 (“nearly every day”). The total score ranges from 0 to 27, with higher scores indicating more severe depressive symptoms. For descriptive analysis of participants' depressive symptom severity, PHQ-9 scores were further categorized into five severity levels: minimal or no depressive symptoms, 0–4; mild depressive symptoms, 5–9; moderate depressive symptoms, 10–14; moderately severe depressive symptoms, 15–19; and severe depressive symptoms, 20–27. A PHQ-9 score of 10 or above was used to indicate moderate-to-severe depressive symptoms. In the present study, the PHQ-9 demonstrated excellent internal consistency (Cronbach's α = 0.938).

#### 2.2.5 Anxiety symptoms

Anxiety symptoms were assessed using the Generalized Anxiety Disorder Scale-7 (GAD-7) (34), a brief and validated self-report tool commonly used in clinical and research settings to screen for generalized anxiety disorder and assess its severity. The GAD-7 consists of seven items measuring key anxiety symptoms, including restlessness, uncontrollable worry, excessive worry about various topics, difficulty relaxing, irritability, and fear. Items are scored based on their frequency over the past two weeks using a four-point Likert scale from 0 (“not at all”) to 3 (“nearly every day”). Total scores range from 0 to 21, with higher scores reflecting greater anxiety severity. For descriptive analysis, GAD-7 scores were categorized into four severity levels: minimal or no anxiety symptoms, 0–4; mild anxiety symptoms, 5–9; moderate anxiety symptoms, 10–14; and severe anxiety symptoms, 15–21. A GAD-7 score of 10 or above was used to indicate moderate-to-severe anxiety symptoms. In this study, the GAD-7 showed excellent internal reliability with a Cronbach's α of 0.955.

### 2.3 Quality control of the survey process

Designated survey sites were established across provinces by collaborating with selected local community health centers or neighborhood committees, ensuring adequate geographic representation. Prior to survey implementation, all investigators received standardized training and were instructed to follow a uniform introductory script. Data were collected through face-to-face, one-on-one interviews.

Participants were recruited by distributing study notices and posting recruitment flyers in community areas. Participant eligibility was rigorously verified based on inclusion and exclusion criteria. The questionnaire was programmed electronically via the Wenjuanxing platform (https://www.wjx.cn/) and accessed by participants through QR code scanning. Each questionnaire was assigned a unique identifier, and informed consent was obtained prior to participation. For participants with preserved cognitive function but limited mobility, trained investigators administered the questions and completed the survey on their behalf.

All surveys were completed and collected on-site. Questionnaires with a completion rate below 80% were excluded. For those with 80%−100% completion, if the missing data were minimal and non-essential, standardized imputation methods were applied; if key items were missing or if the number of missing items was substantial, the questionnaire was discarded. All eligible responses were serialized and archived. Data were automatically consolidated by the Wenjuanxing platform and securely stored on a centralized server for subsequent data management and analysis.

### 2.4 Statistical analysis

All statistical analyses were conducted using Stata version 18.0 and R software. The Shapiro–Wilk test was used to assess the normality of continuous variables. As most variables were not normally distributed, continuous data were summarized using median and interquartile range [M (P25, P75)], and group comparisons were performed using the Mann–Whitney U test. Categorical variables were expressed as counts and percentages [*n* (%)], with between-group differences evaluated using the chi-square test. The severity distributions of depressive and anxiety symptoms were also described using counts and percentages, and occupational differences in symptom severity categories were examined using the chi-square test.

Partial correlation analyses were conducted to assess associations among WFC, FWC, health literacy, and depressive and anxiety symptoms (measured by PHQ-9 and GAD-7 scores), with potential confounders statistically controlled. The controlled covariates included age, gender, marital status, education level, monthly per capita household income, household registration type, place of residence, number of children, chronic disease status, smoking status, and alcohol consumption. Mediation analyses were conducted using R software with a non-parametric bootstrap procedure based on 5,000 resamples. For each occupational group, separate parallel mediation models were estimated for WFC and FWC as independent variables and for anxiety symptoms and depressive symptoms as dependent variables. In each model, the three dimensions of health literacy—health promotion, health care, and disease prevention—were entered simultaneously as parallel mediators. This modeling strategy allowed the indirect association through each health literacy dimension to be estimated while statistically adjusting for the other two dimensions. Given the relatively high intercorrelations among the three health literacy dimensions, this parallel mediation approach was chosen to reduce the risk of overinterpreting dimension-specific indirect associations that might arise from separate single-mediator models. All mediation models were adjusted for age, gender, marital status, education level, monthly income, household registration type, residence, number of children, chronic disease status, smoking status, and alcohol consumption. Because the data were cross-sectional, mediation results were interpreted as indirect associations rather than evidence of causal pathways. All hypothesis tests were conducted as two-tailed, with a significance level set at α = 0.05.

## 3 Results

### 3.1 Descriptive characteristics

As shown in Table 1, the study included a total of 2,487 white-collar workers and 2,104 blue-collar workers. There were no significant differences between the two groups in terms of gender, ethnicity, presence of chronic disease, or alcohol consumption (*P* > 0.05). However, the groups differed significantly across several sociodemographic variables, including age distribution, income level, marital status, household registration type, place of residence, number of children, and smoking status (*P* < 0.05).

**Table 1:** Comparison of demographic characteristics, WFC, FWC, health literacy, and anxiety and depressive symptoms between white- and blue-collar workers.

Variable	White-collar (*n* = 2,487)	Blue-collar (*n* = 2,104)	*x*^2^/z value	*P*-value
Sex, *n* (%)			0.973	0.324
Male	1,307 (52.55)	1,075 (51.09)		
Female	1,180 (47.45)	1,029 (48.91)		
Age group, n (%)			9.564	0.023
18–30	634 (25.49)	549 (26.09)		
31–40	719 (28.91)	525 (24.95)		
41–50	865 (34.78)	780 (37.07)		
51–60	269 (10.82)	250 (11.88)		
Ethnicity, *n* (%)			0.964	0.326
Han	100 (4.02)	97 (4.61)		
Other	2,387 (95.98)	2,007 (95.39)		
Monthly income (RMB), *n* (%)			154.392	< 0.001
≤ 3,000	289 (11.62)	505 (24.00)		
3,001–7,500	1,339 (53.84)	1,118 (53.14)		
>7,500	859 (34.54)	481 (22.86)		
Marital status, *n* (%)			5.054	0.025
Married	2,001 (80.46)	1,636 (77.76)		
Other	486 (19.54)	468 (22.24)		
Household registration type, *n* (%)			222.921	< 0.001
Agricultural	483 (19.42)	829 (39.40)		
Non-agricultural	2,004 (80.58)	1,275 (60.60)		
Residence, *n* (%)			76.181	< 0.001
Rural	293 (11.78)	448 (21.29)		
Urban	2,194 (88.22)	1,656 (78.71)		
Number of children, *n* (%)			24.783	< 0.001
0	750 (30.51)	593 (29.14)		
1	1,205 (49.02)	899 (44.18)		
≥2	503 (20.46)	543 (26.68)		
Chronic disease status, *n* (%)			0.013	0.910
No	2,087 (83.92)	1,763 (83.79)		
Yes	400 (16.08)	341 (16.21)		
Smoking status, *n* (%)			19.310	< 0.001
No	2,133 (85.77)	1,703 (80.94)		
Yes	354 (14.23)	401 (19.06)		
Alcohol consumption, *n* (%)			1.813	0.178
No	1,345 (54.08)	1,096 (52.09)		
Yes	1,142 (45.92)	1,008 (47.91)		
WFC	14.000 (10.0, 16.0)	14.000 (10.0, 15.0)	−1.381	0.167
FWC	11.000 (9.0, 15.0)	12.000 (10.0, 15.0)	−2.981	0.003
Total health literacy, M (P25, P75)	36.000 (36.0, 42.0)	36.000 (34.0, 39.0)	−7.238	< 0.001
Health promotion, M (P25, P75)	12.000 (12.0, 14.0)	12.000 (12.0, 13.0)	−6.701	< 0.001
Health care, M (P25, P75)	12.000 (12.0, 14.0)	12.000 (11.0, 13.0)	−6.310	< 0.001
Disease prevention, M (P25, P75)	12.000 (12.0, 14.0)	12.000 (12.0, 13.0)	−6.178	< 0.001
Depression symptoms, M (P25, P75)	4.000 (1.0, 9.0)	5.000 (1.0, 9.0)	−1.124	0.261
Anxiety symptoms, M (P25, P75)	3.000 (0.0, 7.0)	3.000 (0.0, 7.0)	−0.465	0.642

Specifically, blue-collar workers had higher proportions of agricultural household registration (39.40%), rural residence (21.29%), multiple-child families (26.68%), and smoking (19.06%), and were generally characterized by lower monthly income levels compared to white-collar workers.

Regarding the key study variables, blue-collar workers reported significantly higher FWC scores than white-collar workers (*P* = 0.003). Additionally, blue-collar workers demonstrated significantly lower total health literacy scores and subscale scores across all three dimensions—health promotion, health care, and disease prevention (*P* < 0.001). Notably, despite multiple differences in sociodemographic and key study variables, no statistically significant differences were observed between the two groups in depressive and anxiety symptom scores (*P* > 0.05). Specifically, the median depressive symptom score was 4.000 (1.0, 9.0) among white-collar workers and 5.000 (1.0, 9.0) among blue-collar workers, while the median anxiety symptom score was 3.000 (0.0, 7.0) in both groups. These findings indicate that although blue-collar workers had higher family-to-work conflict and lower health literacy, the overall levels of depressive and anxiety symptoms were comparable between occupational groups.

### 3.2 Severity distribution of anxiety and depressive symptoms among participants

To further describe participants' anxiety and depressive symptom severity, depressive and anxiety symptoms were categorized according to the standard severity cut-off scores of the PHQ-9 and GAD-7. As shown in Table 2, 2,263 participants (49.29%) had minimal or no depressive symptoms, 1,555 (33.87%) had mild depressive symptoms, 415 (9.04%) had moderate depressive symptoms, 281 (6.12%) had moderately severe depressive symptoms, and 77 (1.68%) had severe depressive symptoms. The overall distribution of depressive symptom severity did not differ significantly between white- and blue-collar workers, χ^2^ = 9.393, P = 0.052. Using PHQ-9 ≥ 10 as the threshold, 773 participants (16.84%) had moderate-to-severe depressive symptoms, including 421 white-collar workers (16.93%) and 352 blue-collar workers (16.73%), with no significant occupational difference, χ^2^ = 0.019, *P* = 0.889.

**Table 2:** Severity distribution of depressive and anxiety symptoms among white- and blue-collar workers.

Section	Category	Total sample *n* (%)	White-collar workers *n* (%)	Blue-collar workers *n* (%)	χ^2^ value	*P*-value
Depressive symptoms, PHQ-9	Minimal or none, 0–4	2,263 (49.29%)	1,247 (50.14%)	1,016 (48.29%)	9.393	0.052
	Mild, 5–9	1,555 (33.87%)	819 (32.93%)	736 (34.98%)		
	Moderate, 10–14	415 (9.04%)	218 (8.77%)	197 (9.36%)		
	Moderately severe, 15–19	281 (6.12%)	150 (6.03%)	131 (6.23%)		
	Severe, 20–27	77 (1.68%)	53 (2.13%)	24 (1.14%)		
Depressive symptoms, PHQ-9	PHQ-9 < 10	3,818 (83.16%)	2,066 (83.07%)	1,752 (83.27%)	0.019	0.889
	PHQ-9 ≥ 10	773 (16.84%)	421 (16.93%)	352 (16.73%)		
Anxiety symptoms, GAD-7	Minimal or none, 0–4	2,719 (59.22%)	1,486 (59.75%)	1,233 (58.60%)	10.653	0.014
	Mild, 5–9	1,373 (29.91%)	722 (29.03%)	651 (30.94%)		
	Moderate, 10–14	402 (8.76%)	212 (8.52%)	190 (9.03%)		
	Severe, 15–21	97 (2.11%)	67 (2.69%)	30 (1.43%)		
Anxiety symptoms, GAD-7	GAD-7 < 10	4,092 (89.13%)	2,208 (88.78%)	1,884 (89.54%)	0.607	0.436
	GAD-7 ≥ 10	499 (10.87%)	279 (11.22%)	220 (10.46%)		

PHQ-9, Patient Health Questionnaire-9; GAD-7, Generalized Anxiety Disorder-7. PHQ-9 ≥ 10 indicates moderate-to-severe depressive symptoms; GAD-7 ≥ 10 indicates moderate-to-severe anxiety symptoms. χ^2^ tests were used to compare symptom severity distributions between white- and blue-collar workers.

For anxiety symptoms, 2,719 participants (59.22%) had minimal or no anxiety symptoms, 1,373 (29.91%) had mild anxiety symptoms, 402 (8.76%) had moderate anxiety symptoms, and 97 (2.11%) had severe anxiety symptoms. The overall distribution of anxiety symptom severity differed significantly between white- and blue-collar workers, χ^2^ = 10.653, *P* = 0.014. However, when GAD-7 ≥ 10 was used as the threshold, 499 participants (10.87%) had moderate-to-severe anxiety symptoms, including 279 white-collar workers (11.22%) and 220 blue-collar workers (10.46%), with no significant occupational difference, χ^2^ = 0.607, *P* = 0.436. These results indicate that although the distribution of anxiety severity categories differed by occupational group, the proportions of workers with clinically relevant moderate-to-severe depressive and anxiety symptoms were comparable between white- and blue-collar workers.

### 3.3 Correlation analysis of WFC, FWC, health literacy, and anxiety and depressive symptoms among white- and blue-collar workers

Partial correlation analysis results (Table 3) revealed distinct association patterns between work-family conflict and both health literacy and anxiety and depressive symptoms across white- and blue-collar workers. In both occupational groups, FWC demonstrated stronger negative correlations with health literacy (white-collar: r = −0.143; blue-collar: r = −0.202) than did WFC (white-collar: r = −0.054; blue-collar: r = −0.117).

**Table 3:** Partial correlation matrix of WFC, FWC, health literacy, and anxiety and depressive symptoms among white- and blue-collar workers.

Group	Variable	WFC	FWC	Total health literacy	Health promotion	Health care	Disease prevention	Depression
White–collar workers	WFC	1						
	FWC	0.734^**^	1					
	Total health literacy	−0.054^**^	−0.143^**^	1				
	Health promotion	−0.061^**^	−0.139^**^	0.903^**^	1			
	Health care	−0.030	−0.113^**^	0.910^**^	0.708^**^	1		
	Disease prevention	−0.057^**^	−0.141^**^	0.936^**^	0.785^**^	0.787^**^	1	
	Depression	0.420^**^	0.516^**^	−0.135^**^	−0.114^**^	−0.122^**^	−0.134^**^	1
	Anxiety	0.418^**^	0.509^**^	−0.138^**^	−0.119^**^	−0.118^**^	−0.142^**^	0.857^**^
Blue–collar workers	WFC	1						
	FWC	0.730^**^	1					
	Total health literacy	−0.117^**^	−0.202^**^	1				
	Health promotion	−0.112^**^	−0.193^**^	0.909^**^	1			
	Health care	−0.101^**^	−0.182^**^	0.906^**^	0.706^**^	1		
	Disease prevention	−0.108^**^	−0.181^**^	0.937^**^	0.800^**^	0.779^**^	1	
	Depression	0.378^**^	0.459^**^	−0.169^**^	−0.158^**^	−0.164^**^	−0.142^**^	1
	Anxiety	0.359^**^	0.440^**^	−0.164^**^	−0.157^**^	−0.156^**^	−0.138^**^	0.839^**^

^*^p < 0.05, ^**^p < 0.01.

Notably, the strength of the negative correlation between WFC and total health literacy—as well as its subdimensions—was consistently higher in blue-collar workers. Similarly, FWC showed stronger positive correlations with depressive and anxiety symptoms than WFC in both groups. Among white-collar workers, the correlations between FWC and depressive symptoms (r = 0.516) and anxiety symptoms (r = 0.509) were notably stronger than those among blue-collar workers (depressive symptoms: r = 0.459; anxiety symptoms: r = 0.440).

Regarding health literacy, it was negatively associated with anxiety and depressive symptoms in both groups, with slightly stronger correlations in blue-collar workers (depressive symptoms: r = −0.169; anxiety symptoms: r = −0.164) compared to white-collar workers (depressive symptoms: r = −0.135; anxiety symptoms: r = −0.138). Overall, these findings indicate that higher levels of WFC and FWC were associated with more severe anxiety and depressive symptoms, whereas higher health literacy was associated with fewer anxiety and depressive symptoms. The correlation patterns also suggest that the associations among work-family conflict, health literacy, and anxiety and depressive symptoms differed to some extent by occupational group.

### 3.4 Parallel mediating role of health literacy dimensions in the association between WFC and anxiety and depressive symptoms

In the following mediation analyses, health promotion, health care, and disease prevention literacy were entered simultaneously as parallel mediators. Therefore, each reported indirect association represents the dimension-specific indirect association after accounting for the other two health literacy dimensions.

As shown in Table 4, among white-collar workers, WFC was positively associated with anxiety symptoms (β = 0.405, *p* < 0.001) and depressive symptoms (β = 0.493, *p* < 0.001). WFC was also negatively associated with the health promotion (β = −0.026, *p* = 0.003) and disease prevention (β = −0.025, *p* = 0.005) subdimensions of health literacy, indicating that higher levels of conflict were associated with lower levels of these health literacy dimensions.

**Table 4:** Parallel mediation analysis of health literacy dimensions in the association between WFC and anxiety and depressive symptoms among white-collar workers.

Pathway	Effect	95% CI		SE	z/t value	*p*-value
		Lower	Upper			
WFC → Anxiety Symptoms	0.405	0.370	0.440	0.018	22.71	< 0.001
WFC → Health promotion	−0.026	−0.043	−0.009	0.009	−2.987	0.003
WFC → Disease prevention	−0.025	−0.043	−0.008	0.009	−2.818	0.005
WFC → Health care	−0.014	−0.032	0.004	0.009	−1.488	0.137
Health promotion → Anxiety Symptoms	0.019	−0.117	0.155	0.069	0.275	0.783
Health care → Anxiety Symptoms	−0.132	−0.259	−0.004	0.065	−2.021	0.043
Disease prevention → Anxiety Symptoms	−0.151	−0.300	−0.001	0.076	−1.976	0.048
WFC → Health promotion → Anxiety Symptoms	0	−0.005	0.004	0.002	−0.234	0.815
WFC → Disease prevention → Anxiety Symptoms	0.004	0	0.010	0.003	1.386	0.166
WFC → Health care → Anxiety Symptoms	0.002	−0.001	0.007	0.002	0.956	0.339
WFC → Depression Symptoms	0.493	0.45	0.536	0.022	22.603	< 0.001
WFC → Health promotion	−0.026	−0.043	−0.009	0.009	−2.987	0.003
WFC → Disease prevention	−0.025	−0.043	−0.008	0.009	−2.818	0.005
WFC → Health care	−0.014	−0.032	0.004	0.009	−1.488	0.137
Health promotion → Depression Symptoms	0.019	−0.147	0.185	0.085	0.227	0.821
Disease prevention → Depression Symptoms	−0.264	−0.446	−0.081	0.093	−2.829	0.005
Health care → Depression Symptoms	−0.085	−0.241	0.071	0.08	−1.064	0.288
WFC → Health promotion → Depression Symptoms	0	−0.005	0.004	0.002	−0.233	0.816
WFC → Disease prevention → Depression Symptoms	0.007	0	0.013	0.003	2.031	0.042
WFC → Health care → Depression Symptoms	0.001	−0.001	0.005	0.001	0.784	0.433

Further analysis revealed that specific health literacy dimensions were negatively associated with anxiety and depressive symptoms: both the health care and disease prevention subdimensions were significantly associated with lower anxiety symptoms, while disease prevention was also significantly associated with lower depressive symptoms. Among all tested mediation pathways, only the indirect path “WFC → disease prevention → depressive symptoms” was statistically significant (β = 0.007, *p* = 0.042), indicating a significant indirect association through disease prevention literacy.

As shown in Table 5, among blue-collar workers, WFC was positively associated with anxiety symptoms (β = 0.359, *p* < 0.001) and depressive symptoms (β = 0.418, *p* < 0.001), and was negatively associated with all three dimensions of health literacy: health promotion (β = −0.053), disease prevention (β = −0.052), and health care (β = −0.050), all with *p* < 0.001. Regarding the associations between health literacy dimensions and anxiety symptoms, health promotion (β = −0.157, *p* = 0.032) and disease prevention (β = −0.213, *p* = 0.001) were significantly associated with lower anxiety symptoms, whereas health care was not statistically significant (β = 0.084, *p* = 0.303). For depressive symptoms, health promotion (β = −0.218, *p* = 0.016) and health care (β = −0.226, *p* = 0.006) were significantly associated with lower depressive symptoms, whereas disease prevention was not statistically significant (β = 0.099, *p* = 0.323).

**Table 5:** Parallel mediation analysis of health literacy dimensions in the association between WFC and anxiety and depressive symptoms among blue-collar workers.

Pathway	Effect	95% CI		SE	z/t value	*p*-value
		Lower	Upper			
WFC → Anxiety Symptoms	0.359	0.320	0.399	0.020	17.694	< 0.001
WFC → Health promotion	−0.053	−0.073	−0.032	0.010	−5.037	< 0.001
WFC → Disease prevention	−0.052	−0.073	−0.031	0.011	−4.862	< 0.001
WFC → Health care	−0.050	−0.071	−0.028	0.011	−4.542	< 0.001
Health promotion → Anxiety Symptoms	−0.157	−0.300	−0.014	0.073	−2.149	0.032
Health care → Anxiety Symptoms	0.084	−0.075	0.242	0.081	1.031	0.303
Disease prevention → Anxiety Symptoms	−0.213	−0.344	−0.082	0.067	−3.186	0.001
WFC → Health promotion → Anxiety Symptoms	0.008	−0.001	0.02	0.005	1.556	0.120
WFC → Disease prevention → Anxiety Symptoms	−0.004	−0.015	0.004	0.005	−0.903	0.366
WFC → Health care → Anxiety Symptoms	0.011	0.003	0.02	0.004	2.419	0.016
WFC → Depression Symptoms	0.418	0.369	0.467	0.025	16.632	< 0.001
WFC → Health promotion	−0.053	−0.073	−0.032	0.010	−5.037	< 0.001
WFC → Disease prevention	−0.052	−0.073	−0.031	0.011	−4.862	< 0.001
WFC → Health care	−0.05	−0.071	−0.028	0.011	−4.542	< 0.001
Health promotion → Depression Symptoms	−0.218	−0.395	−0.041	0.090	−2.415	0.016
Disease prevention → Depression Symptoms	0.099	−0.097	0.295	0.100	0.988	0.323
Health care → Depression Symptoms	−0.226	−0.388	−0.064	0.083	−2.731	0.006
WFC → Health promotion → Depression Symptoms	0.011	0.001	0.021	0.005	2.216	0.027
WFC → Disease prevention → Depression Symptoms	−0.005	−0.014	0.004	0.004	−1.176	0.240
WFC → Health care → Depression Symptoms	0.011	0.002	0.018	0.004	2.753	0.006

Mediation analysis indicated that the “WFC → health care → anxiety symptoms” path constituted a significant indirect association (β = 0.011, *p* = 0.016). In addition, two indirect pathways were significant in relation to depressive symptoms: “WFC → health promotion → depressive symptoms” (β = 0.011, *p* = 0.027) and “WFC → health care → depressive symptoms” (β = 0.011, *p* = 0.006). No significant indirect associations were observed through disease prevention.

### 3.5 Parallel mediating role of health literacy dimensions in the association between FWC and anxiety and depressive symptoms

As shown in Table 6, among white-collar workers, FWC was positively associated with anxiety symptoms (β = 0.507, *p* < 0.001) and depressive symptoms (β = 0.609, *p* < 0.001). FWC also was negatively associated with all three dimensions of health literacy: health promotion (β = −0.061), disease prevention (β = −0.064), and health care (β = −0.053), all with *p* < 0.001. Regarding the associations between health literacy dimensions and anxiety symptoms, none of the three dimensions showed a statistically significant association. For depressive symptoms, only the disease prevention subdimension demonstrated a statistically significant negative association (β = −0.206, *p* = 0.020). Mediation analysis revealed no significant indirect associations for anxiety symptoms. However, for depressive symptoms, the pathway “FWC → disease prevention → depressive symptoms” showed a statistically significant indirect association (β = 0.013, *p* = 0.017), suggesting a dimension-specific indirect association through disease prevention literacy.

**Table 6:** Parallel mediation analysis of health literacy dimensions in the association between FWC and anxiety and depressive symptoms among white-collar workers.

Pathway	Effect	95% CI		SE	z/t value	*p*-value
		Lower	Upper			
FWC → Anxiety Symptoms	0.507	0.472	0.541	0.017	29.052	< 0.001
FWC → Health promotion	−0.061	−0.078	−0.044	0.009	−6.942	< 0.001
FWC → Disease prevention	−0.064	−0.081	−0.046	0.009	−7.011	< 0.001
FWC → Health care	−0.053	−0.071	−0.034	0.009	−5.620	< 0.001
Health promotion → Anxiety Symptoms	0.059	−0.069	0.188	0.066	0.904	0.366
Health care → Anxiety Symptoms	−0.102	−0.244	0.039	0.072	−1.415	0.157
Disease prevention → Anxiety Symptoms	−0.099	−0.220	0.022	0.062	−1.607	0.108
FWC → Health promotion → Anxiety Symptoms	−0.004	−0.013	0.005	0.005	−0.756	0.449
FWC → Disease prevention → Anxiety Symptoms	0.007	−0.003	0.017	0.005	1.252	0.210
FWC → Health care → Anxiety Symptoms	0.005	−0.001	0.013	0.004	1.456	0.145
FWC → Depression Symptoms	0.609	0.567	0.651	0.021	28.438	< 0.001
FWC → Health promotion	−0.061	−0.078	−0.044	0.009	−6.942	< 0.001
FWC → Disease prevention	−0.064	−0.081	−0.046	0.009	−7.011	< 0.001
FWC → Health care	−0.053	−0.071	−0.034	0.009	−5.620	< 0.001
Health promotion → Depression Symptoms	0.067	−0.091	0.225	0.081	0.830	0.407
Disease prevention → Depression Symptoms	−0.206	−0.381	−0.032	0.089	−2.322	0.020
Health care → Depression Symptoms	−0.045	−0.194	0.104	0.076	−0.594	0.552
FWC → Health promotion → Depression Symptoms	−0.004	−0.013	0.006	0.005	−0.829	0.407
FWC → Disease prevention → Depression Symptoms	0.013	0.001	0.022	0.005	2.391	0.017
FWC → Health care → Depression Symptoms	0.002	−0.005	0.010	0.004	0.669	0.503

As shown in Table 7, among blue-collar workers, FWC was positively associated with anxiety symptoms (β = 0.431, *p* < 0.001) and depressive symptoms (β = 0.506, *p* < 0.001). FWC also was negatively associated with all three dimensions of health literacy: health promotion (β = −0.090), disease prevention (β = −0.086), and health care (β = −0.088), all with *p* < 0.001. Regarding the associations between health literacy dimensions and anxiety symptoms, disease prevention was significantly associated with lower anxiety symptoms (β = −0.165, *p* = 0.011), whereas health promotion (β = −0.098, *p* = 0.166) and health care (β = 0.083, *p* = 0.290) were not statistically significant. For depressive symptoms, health care showed a statistically significant negative association (β = −0.169, *p* = 0.035), while health promotion showed a marginal association with lower depressive symptoms (β = −0.148, *p* = 0.091), and disease prevention was not statistically significant (β = 0.098, *p* = 0.311).

**Table 7:** Parallel mediation analysis of health literacy dimensions in the association between FWC and anxiety and depressive symptoms among blue-collar workers.

Pathway	Effect	95% CI		SE	z/t value	*p*-value
		Lower	Upper			
FWC → Anxiety Symptoms	0.431	0.393	0.470	0.020	22.034	< 0.001
FWC → Health promotion	−0.090	−0.110	−0.070	0.010	−8.846	< 0.001
FWC → Disease prevention	−0.086	−0.106	−0.065	0.010	−8.274	< 0.001
FWC → Health care	−0.088	−0.109	−0.067	0.011	−8.299	< 0.001
Health promotion → Anxiety Symptoms	−0.098	−0.236	0.041	0.071	−1.385	0.166
Health care → Anxiety Symptoms	0.083	−0.070	0.236	0.078	1.058	0.290
Disease prevention → Anxiety Symptoms	−0.165	−0.291	−0.038	0.065	−2.547	0.011
FWC → Health promotion → Anxiety Symptoms	0.009	−0.006	0.025	0.008	1.094	0.274
FWC → Disease prevention → Anxiety Symptoms	−0.007	−0.023	0.007	0.008	−0.922	0.356
FWC → Health care → Anxiety Symptoms	0.015	0.003	0.028	0.006	2.314	0.021
FWC → Depression Symptoms	0.506	0.459	0.554	0.024	20.853	< 0.001
FWC → Health promotion	−0.090	−0.110	−0.070	0.010	−8.846	< 0.001
FWC → Disease prevention	−0.086	−0.106	−0.065	0.010	−8.274	< 0.001
FWC → Health care	−0.088	−0.109	−0.067	0.011	−8.299	< 0.001
Health promotion → Depression Symptoms	−0.148	−0.320	0.023	0.087	−1.693	0.091
Disease prevention → Depression Symptoms	0.098	−0.092	0.288	0.097	1.014	0.311
Health care → Depression Symptoms	−0.169	−0.326	−0.012	0.080	−2.106	0.035
FWC → Health promotion → Depression Symptoms	0.013	−0.003	0.027	0.008	1.710	0.087
FWC → Disease prevention → Depression Symptoms	−0.008	−0.021	0.006	0.007	−1.212	0.225
FWC → Health care → Depression Symptoms	0.015	0.001	0.024	0.006	2.486	0.013

Mediation analysis indicated that the pathways “FWC → health care → anxiety symptoms” (β = 0.015, *p* = 0.021) and “FWC → health care → depressive symptoms” (β = 0.015, *p* = 0.013) constituted significant indirect associations. The indirect path “FWC → health promotion → depressive symptoms” (β = 0.013, *p* = 0.087) demonstrated a marginally significant indirect association. No significant indirect associations were observed through disease prevention.

## 4 Discussion

This study examined the associations of WFC and FWC with anxiety and depressive symptoms among white- and blue-collar workers, with a particular focus on the parallel mediating role of health literacy dimensions. The results revealed significant occupational differences in conflict patterns and health literacy levels. Blue-collar workers exhibited higher levels of FWC than white-collar workers (*p* = 0.003), consistent with the findings of Major et al. (14), who suggested that individuals with limited work schedule flexibility tend to experience more FWC. Blue-collar occupations are generally associated with more rigid schedules, leaving little room to accommodate family demands—a pattern also confirmed by Pak et al. (13). Additionally, blue-collar workers scored significantly lower in overall and dimensional health literacy (*p* < 0.001), aligning with Lindert et al. (29), who reported a strong link between socioeconomic status and health literacy.

An important descriptive finding of this study was that white- and blue-collar workers showed comparable levels of clinically relevant anxiety and depressive symptoms. Specifically, 16.84% of the total sample had moderate-to-severe depressive symptoms, and 10.87% had moderate-to-severe anxiety symptoms. Although the distribution of anxiety severity categories differed significantly between occupational groups, the proportions of workers with GAD-7 scores ≥10 did not differ significantly between white- and blue-collar workers. Similarly, neither the overall distribution of depressive symptom severity nor the proportion of workers with PHQ-9 scores ≥10 showed significant occupational differences. These findings suggest that occupational category alone may not directly translate into different levels of anxiety and depressive symptoms. Instead, white- and blue-collar workers may experience comparable symptom burdens through different associated pathways. For white-collar workers, cognitive overload, blurred work-family boundaries, and performance pressure may be associated with greater psychological vulnerability, whereas for blue-collar workers, lower health literacy, limited schedule flexibility, and restricted access to health resources may constitute important correlates of anxiety and depressive symptoms. Therefore, the absence of significant occupational differences in moderate-to-severe anxiety and depressive symptoms does not weaken the study findings; rather, it highlights the importance of examining occupation-specific indirect associations.

The findings support the widely documented positive association between work-family conflict and anxiety and depressive symptoms. Frone (8) found that work-family conflict is significantly associated with psychiatric disorders, including depression and anxiety. Sugawara et al. (10) also identified work-family conflict as a crucial mediating variable between job stress and psychological outcomes. From a neuroscientific perspective, Golkar et al. (5) demonstrated that prolonged occupational stress alters emotional regulation and neural connectivity, which may compromise psychological wellbeing. This study further highlights occupational disparities in these associations, echoing Byron's (11) meta-analysis, which identified occupational characteristics as key moderators of work-family conflict and its outcomes.

Compared to prior studies (24, 25), this research emphasizes the stratifying role of occupation. Among blue-collar workers, the negative correlation between WFC and health literacy was generally stronger, while white-collar workers showed a more pronounced positive correlation between FWC and anxiety and depressive symptoms. This suggests that while WFC and FWC are widespread stressors, their associations with health literacy and anxiety and depressive symptoms may vary significantly between groups—consistent with Michel et al. (20), who noted that both antecedents and consequences of work-family conflict differ across contexts and populations.

Among white-collar workers, WFC was indirectly associated with depressive symptoms primarily through lower disease prevention literacy. This may reflect the high-pressure, high-responsibility nature of cognitive work: when work infringes on personal time, individuals may have fewer opportunities to attend to preventive healthcare and early warning signs, which may be linked to greater depressive symptom severity. Knowledge-based jobs typically require sustained cognitive engagement and may blur the work-family boundary, thereby being associated with psychological strain—consistent with role stress theory (35) and the burnout model proposed by Lee and Ashforth (7). In contrast, among blue-collar workers, WFC was indirectly associated with anxiety and depressive symptoms through specific health literacy dimensions, particularly health care and health promotion literacy. As physically demanding roles rely heavily on physical fitness, WFC may be associated with limited access to or lower engagement in health-promoting behaviors (e.g., exercise, diet) and medical care. This aligns with findings by Yi et al. (6) on the relationship between job stress, psychological wellbeing, and work ability in coal chemical workers.

The indirect associations involving FWC also differed by occupational group. Among white-collar workers, disease prevention literacy mediated the association between FWC and depressive symptoms, but no significant indirect association was observed for anxiety symptoms. This finding suggests that when family responsibilities interfere with work, white-collar workers may report more depressive symptoms when their ability to recognize health risks and engage in preventive behaviors is lower. Among blue-collar workers, health care literacy mediated the associations of FWC with both anxiety and depressive symptoms. This pattern may reflect the greater relevance of practical access to health services, health consultation, and appropriate help-seeking among workers with fewer socioeconomic and workplace resources. These findings indicate that health literacy should not be treated as a single homogeneous construct; instead, different dimensions of health literacy may show distinct indirect associations in different occupational groups and conflict directions.

Theoretically, this study integrates work-family conflict theory, health literacy theory, and occupational stratification into a comprehensive explanatory framework. First, it extends the applicability of the model proposed by Greenhaus and Beutell (12), demonstrating that while WFC and FWC are associated with anxiety and depressive symptoms among both cognitive and manual workers, their patterns of indirect association differ. This finding is consistent with the job demands-resources model and spillover theory employed by Abdou et al. (9) in their analysis of work stress, work-family conflict, and psychological distress. Second, by introducing health literacy as a mediator, this study advances beyond prior research focused solely on direct associations or stress as the intermediary (15, 17). Previous findings by Wang et al. (30) demonstrated that health literacy predicts resilience in college students; our results extend this concept to working populations experiencing WFC and FWC.

Furthermore, the occupational classification lens highlights how socioeconomic status and job characteristics may shape work-family conflict experiences and their associations with anxiety and depressive symptoms, contributing to the literature on social determinants of health inequalities. This is in line with Chandler's (2) argument that work-family conflict should be considered a public health issue. Nasharudin and Rui (18) also demonstrated that psychosocial safety climate affects psychological health via work-family conflict, with varied needs across occupational groups. These insights may inform work-family policy development, underscoring the necessity of occupation-specific strategies.

Differentiated interventions may be considered based on occupational characteristics. For white-collar workers, emphasis could be placed on enhancing disease prevention awareness, recognizing early signs of anxiety and depressive symptoms, and encouraging professional help-seeking. As Kim et al. (3) suggested, the spillover associations of WFC with work attitudes deserve attention; employers may address these issues by offering flexible work arrangements and psychological support. For blue-collar workers, interventions could prioritize improving access to healthcare and promoting healthy lifestyles through convenient medical services and support systems. Tsang et al. (16) reported that work engagement and self-efficacy mediate the relationship between FWC and work productivity—suggesting that these factors could be targeted among blue-collar groups. Additionally, increasing schedule flexibility and family support (e.g., childcare services) may be relevant to FWC in manual workers, consistent with findings from Su and Jiang (19) on female university faculty in China. Organizations should avoid one-size-fits-all solutions and tailor policies to occupational group needs, thereby enhancing both effectiveness and workplace health equity. This aligns with Luo's (4) recommendation regarding the mediating roles of social support and self-efficacy in the WFC–psychological wellbeing relationship.

This study has several limitations. First, its cross-sectional design precludes causal inference and cannot rule out reverse causality—i.e., anxiety and depressive symptoms may influence perceived WFC/FWC or health literacy. Therefore, the mediation results should be interpreted as indirect associations rather than evidence of causal pathways or temporal ordering. Second, despite the large sample size, selection bias may be present, especially if blue-collar individuals with low health literacy were underrepresented. Third, self-reported measures may be subject to bias, particularly in assessing conflict and anxiety and depressive symptoms. Fourth, the binary occupational classification (cognitive vs. manual) may overlook intra-group heterogeneity—for instance, different subtypes of cognitive (e.g., managerial, professional, clerical) or manual (e.g., agricultural, manufacturing, service) work may vary substantially. Lastly, potential moderating factors such as job nature and organizational culture were not considered.

Future research should adopt longitudinal designs to explore the dynamic trajectories of WFC, health literacy, and anxiety and depressive symptoms, enabling stronger causal claims. Occupational categories should be further refined to identify nuanced subgroup differences. Including other potential mediators/moderators—such as social support, coping strategies, organizational culture, and policy environment—would offer a more comprehensive understanding of the WFC–health literacy–anxiety and depressive symptoms nexus. Evaluating the effectiveness of tailored health literacy interventions across occupational settings could also provide actionable insights. Importantly, understanding how organizational-level strategies can jointly promote employee health literacy and work-family balance may prove vital in safeguarding workers' psychological wellbeing.

## 5 Conclusion

This study found that both WFC and FWC were positively associated with anxiety and depressive symptoms among white- and blue-collar workers, though the patterns of indirect association varied by occupational group. Blue-collar workers reported higher FWC and lower health literacy than white-collar workers, whereas the two groups showed comparable proportions of moderate-to-severe depressive and anxiety symptoms. Health literacy may play distinct roles across occupational groups: among white-collar workers, the indirect association was primarily observed through the disease prevention dimension, while for blue-collar workers, the indirect associations were more pronounced through the health care and health promotion dimensions.

These findings underscore the necessity of adopting differentiated strategies in the development of work-family balance policies and workplace programs addressing anxiety and depressive symptoms. Tailored interventions that address the specific characteristics and needs of various occupational groups are essential to improving psychological wellbeing in the workforce. In particular, disease prevention literacy may be more relevant for white-collar workers, whereas health care literacy and health promotion literacy may be more important intervention targets for blue-collar workers. Future workplace health programs should combine work-family balance support with occupation-specific health literacy promotion to address anxiety and depressive symptoms associated with work-family role conflicts. Given the cross-sectional design, these findings should be interpreted as associations rather than evidence of causal pathways, and future longitudinal or intervention studies are needed to clarify the temporal ordering among work-family conflict, health literacy, and anxiety and depressive symptoms.

## Data Availability

The raw data supporting the conclusions of this article will be made available by the authors, without undue reservation.
